# Enhanced Antibacterial Activity of *Acinetobacter baumannii* Bacteriophage ØABP-01 Endolysin (LysABP-01) in Combination with Colistin

**DOI:** 10.3389/fmicb.2016.01402

**Published:** 2016-09-07

**Authors:** Rapee Thummeepak, Thawatchai Kitti, Duangkamol Kunthalert, Sutthirat Sitthisak

**Affiliations:** ^1^Department of Microbiology and Parasitology, Faculty of Medical Science, Naresuan University, PhitsanulokThailand; ^2^Faculty of Oriental Medicine, Chiang Rai College, Chiang RaiThailand; ^3^Centre of Excellence in Medical Biotechnology, Faculty of Medical Science, Naresuan University, PhitsanulokThailand

**Keywords:** bacteriophage, *Acinetobacter baumannii*, endolysin, colistin

## Abstract

Endolysins are lytic enzymes produced by bacteriophages with their ability to degrade the cell wall of bacterial hosts. Endolysin (LysABP-01) from *Acinetobacter baumannii* bacteriophage ØABP-01 was cloned, overexpressed and characterized. Endolysin LysABP-01 has a globular structure consisting of lysozyme-like (*N*-acetyl-β-D-muramidase) catalytic domain. It contains 185 amino acids which correspond to a 21.1 kDa protein. The lytic activity of the recombinant endolysin protein was determined by a plate lysis assay for its ability to lyse the autoclaved cell (crude cell wall) of the different bacterial species. LysABP-01 can degrade the crude cell wall of *A. baumannii* strains, *Escherichia coli* and *Pseudomonas aeruginosa* but not of *Staphylococcus aureus*. The antibacterial activity of LysABP-01 and its synergism with various antibiotics were tested. The results exhibited elevated antibacterial activity in a combination of the sub-MIC LysABP-01 and colistin. The checkerboard assay for measuring antibiotic synergy of LysABP-01 and colistin was performed. This combination was synergistic against various drug-resistant strains of *A. baumannii* (FIC index < 0.5). In summary, our study highlights the ability of LysABP-01 endolysin to hydrolyze the *A. baumannii* cell wall and its synergistic interaction with colistin.

## Introduction

*Acinetobacter baumannii* has emerged as a clinically significant pathogen that resistance to most available antibiotics. The increasing prevalence of multidrug-resistant *A. baumannii* (MDRAB) and extensively drug-resistant *A. baumannii* (XDRAB) infections has been reported worldwide (Perez et al., 2007). To date, colistin and tigecycline remain the most active antibiotics against drug-resistant *A. baumannii* (Henwood et al., 2002; Falagas and Kasiakou, 2005). However, the utilization of these drugs is limited due to high rates of toxicity and development of resistance (Falagas and Kasiakou, 2005; Navon-Venezia et al., 2007; Cai et al., 2012). Therefore, alternative antibacterial agents for treatments of MDRAB and XDRAB are urgently required.

Endolysins are lytic enzymes produced by bacteriophages during the last step of their replicative cycle. The enzymes degrade the cell wall of bacterial hosts and lead to cell lysis and phage progeny release (Young, 1992). Endolysins are classified as a new class of antimicrobials for the treatment of drug-resistant bacterial infection because of their rapid action, low evidence of resistance development and low cytotoxicity against mammalian cells (Schmelcher et al., 2012). A number of studies have demonstrated the efficiency of recombinant phage endolysins against gram-positive pathogens *in vitro* and in animal models. In contrast, applications of endolysins specific to gram-negative bacteria are limited because the outer cell membrane (OM) prevents exogenously applied endolysins from attracting the peptidoglycan layer (Fischetti, 2010; Schmelcher et al., 2012). Thus, many studies have focused on the enhancement of OM permeability using chelators, weak organic acids or high hydrostatic pressure (Briers et al., 2008, 2011; Oliveira et al., 2014, 2016). The chelator EDTA is very useful as an OM permeabilizer because of its ability to destabilize the lipopolysaccharide structure (Briers et al., 2011). However, the potential for future applications of an endolysin-EDTA combination is limited only for the topical treatment of localized bacterial infections, such as burn wound, eye and ear infections. The use of this combination is not suitable for the treatment of systemic infections as EDTA inhibits the blood clotting at the low concentration (Triantaphpyllopoulos et al., 1955). Combinations of the lytic enzyme with other antibacterials may produce synergistic effects or reduce the dose of a single agent (Djurkovic et al., 2005).

We previously characterized ØABP-01, a lytic phage which infects MDRAB strains (Kitti et al., 2014). We found that the ØABP-01 genome contains the endolysin encoding gene. In this study, we examined the antibacterial activity of LysABP-01 alone and in combination with conventional antibiotics against both MDRAB and XDRAB strains.

## Materials and Methods

### Bacteriophage, Bacterial Strains, Plasmids and Growth Conditions

Bacteriophage, bacterial strains and plasmids used in this study are listed in **Table 1**. *A. baumannii* strain AB 1589 was used as the host for the ØABP-01 phage propagation. Four additional isolates were collected from difference hospitals in Thailand (**Table 2**). All isolates were identified on the species level by using biochemical tests and the PCR detection of the *bla*_OXA-51_ gene (Brown et al., 2005). All bacterial strains were grown in Luria-Bertani (LB) broth or agar (Hardy Diagnostics, Santa Maria, CA, USA) at 37°C.

**Table 1:** Bacteriophage, bacterial strains, plasmids and primers used in this study.

Strains, phage, plasmids, or primers	Relevant characteristic(s), function, or sequence	Source or reference
**Bacteriophage**		
ØABP-01	*A. baumannii* phage from the waste water treatment plant	Kitti et al., 2014
**Bacterial strains**		
ATCC 19606	*A. baumannii* reference strain	ATCC
AB 1589	MDRAB strain, host for ØABP-01	Kitti et al., 2015
DH5∝	*E. coli* strain used for cloning	Novagen, Germany
BL21 (DE3) pLysS	*E. coli* strain used for protein overexpression, Cam^r^	Novagen, Germany
ATCC 25922	*E. coli* reference strain	ATCC
ATCC 27853	*Ps. aeruginosa* reference strain	ATCC
ATCC 6538	*S. aureus* reference strain	ATCC
**Plasmids**		
pBluescript II	DNA cloning and sequencing vector, Amp^r^	Fermentas, USA
pRSETa	Overexpression vector, Amp^r^	Invitrogen, USA
pBluescript-*lysABP-01*	pBluescript II containing the *lysABP-01*	This study
pRSET-*lysABP-01*	pRSETa containing the *lysABP-01*	This study
**Primers**		
EndolysinABP-F	5′-GC*GGATCC*ATGATTCTGACTAAAGACGGATTTAGTATT-3′	This study
EndolysinABP-R	5′-GC*GAATTC*CTATAAGCTCCGTAGAGCACGTTC-3′	
*bla*_OXA-51_-F	5′-TAATGCTTTGATCGGCCTTG-3′	Brown et al., 2005
*bla*_OXA-51_-R	5′-TGGATTGCACTTCATCTTGG-3’	

**Table 2:** Characteristics of clinical *A. baumannii* strains and their susceptibility to the LysABP-01 alone and in combination with colistin.

Strains ID	Year of isolation/Hospital	Drug resistance type	MIC_colistin_ (μg/ml)		MIC_LysABP-01_ (μM)		FICI
			Alone	Combined	Alone	Combined	
AB 1589	2007/H1	MDRAB	1	0.125	20	1.250	0.188
AB 0022	2013/H2	XDRAB	2	0.250	20	1.250	0.188
AB 0140	2014/H3	XDRAB	1	0.125	10	0.3125	0.156
AB 0269	2014/H4	XDRAB	1	0.125	10	0.625	0.188
AB 0405	2015/H5	XDRAB	0.5	0.0625	10	0.3125	0.156

### Cloning of *lysABP-01*

Bacteriophage ØABP-01 genomic DNA was prepared according to the method reported in our previous study (Kitti et al., 2014). The endolysin gene *lysABP-01* (accession no. KF548002) was PCR amplified by using the phage DNA as a template with primers EndolysinABP-F and EndolysinABP-R (**Table 1**) which generated a 558 bp PCR product flanked by the restriction sites *Bam*HI and *Eco*RI. The resulting PCR product was cut with *Bam*HI/*Eco*RI restriction enzymes and cloned into the plasmid pBluescript II (pBluescript-*lysABP-01*). The *lysABP-01* in the pBluescript II was then subcloned into pRSETa expression vector (pRSET-*lysABP-01*). All initial DNA cloning procedures were carried out in *Escherichia coli* DH5α and then transformed into *E. coli* BL21 (DE3) pLysS. Restriction digestions and sequencing were used to verify the integrity of the cloned fragment.

### Overexpression and Purification of LysABP-01

Log phase culture of *E. coli* BL21 (DE3) pLysS containing pRSET-*lysABP-01* (A_600_ ~ 0.5) was induced by the addition of IPTG to the final concentration of 1 mM. After incubation for 4 h at 37°C, cells were pelleted, washed and frozen at -80°C. The expressed protein was found in the insoluble cellular fraction, and was thus purified from the inclusion bodies. Thawed cells were resuspended in lysis buffer (145 mM NaCl, 20 mM Tris-Cl, pH 7.4) and disrupted by sonication with a ultrasonic cell disrupter (Sonic & Material Inc, Newtown, CT, USA). The soluble fraction was removed by centrifugation (5,000 × *g*). The pellets containing the inclusion bodies were solubilized in binding buffer containing urea (6 M urea, 500 mM NaCl, 20 mM Tris-Cl, 5 mM imidazole, pH 7.9). The solubilized recombinant protein was purified on the affinity chromatography column (His⋅Bind Kits, Novagen, Germany) under denaturing condition according to the manufacturer’s instructions. The purified protein fractions were pooled and then diluted with four times volume of renaturation buffer (PBS containing 0.5 mM PMSF, 0.3 M arginine, 1% glycerol). The diluted protein was dialyzed against dialysis buffer (PBS containing 1 M urea, 1% glycerol) for overnight and then dialyzed against storage buffer (PBS containing 1% glycerol). The protein obtained after dialysis was concentrated using the vivaspin500 concentrator (GE Healthcare, UK). The purity of protein was checked by 12% SDS-PAGE, followed by staining of the SDS-PAGE gels with coomassie brilliant blue G-250 and the Pierce 6xHis Protein Tag Stain Reagent Set (Thermo sciencetific, USA). Protein concentrations were determined by the Bio-Rad protein assay (BioRad, Hercules, CA, USA).

### Antibiotic Susceptibility Testing

The disk diffusion method was performed for all *A. baumannii* isolates to determine the susceptibility of amikacin (30 μg), cefotaxime (30 μg), ceftazidime (30 μg), ceftriaxone (30 μg), cefepime (30 μg), ciprofloxacin (5 μg), gentamicin (10 μg), imipenem (10 μg), meropenem (10 μg), trimethoprim/ulfamethoxazole (1.25/23.75 μg), tetracycline (30 μg), cefoperazone/sulbactam (105 μg), piperacillin/tazobactam (100/10 μg), colistin (10 μg) and tigecycline (15 μg) (Oxoid discs, UK). The results were interpreted according to the Clinical and Laboratory Standards Institute (CLSI) guidelines (Clinical and Laboratory Standards Institute [CLSI], 2014). The antibiotic resistance profile was classified as multidrug-resistant *A. baumannii* (MDRAB) or extensively drug-resistant *A. baumannii* (XDRAB) based on a previously published description (Magiorakos et al., 2012).

### Minimum Inhibitory Concentration (MIC)

Minimum inhibitory concentrations was determined by the CLSI recommended broth microdilution techniques in Mueller-Hinton broth (HiMedia) (Clinical and Laboratory Standards Institute [CLSI], 2014). The antibiotics; ciprofloxacin, imipenem, colistin, chloramphenicol, gentamycin, erythromycin and tetracycline obtained from Sigma–Aldrich (St. Louis, MO, USA) were used. The MIC was defined as the lowest concentration of antibiotic that inhibit the bacterial growth. All MIC experiments were performed in duplicate and used *E. coli* ATCC 25922 as a quality control strain. The MIC value was used to calculate sub-MIC values (0.25 × the MIC) for the next experiment.

### Plate Lysis Assay

Plate lysis assay was performed as described previously (Donovan et al., 2006) with some modifications. Bacterial cells of *A. baumannii* ATCC 19606, *A. baumannii* AB 1589, *Escherichia coli* ATCC 25922, *Pseudomonas aeruginosa* ATCC 27853 or *Staphylococcus aureus* ATCC 6538 in mid log phase were collected, washed once and suspended in PBS. The suspension was then autoclaved and centrifuged. The resulting pellet was resuspended in PBS (2% [vol/vol] of initial culture volume) and used as the substrate. Ten microliters of purified LysABP-01 (10 μM) was spotted onto the agar plate (1.5%) containing the substrate (5%). An equal volume of 10 μM egg white lysozyme was used as a positive control whereas PBS and storage buffer were used as negative controls. The spotted plates were incubated at room temperature.

### Antibacterial Activity

Bacterial growth inhibition assay was performed as described previously (Knezevic and Petrovic, 2008) with little modifications. Bacterial colonies were transferred to Mueller Hilton broth (HiMedia) and cultured at 37°C for 4 h to reach mid-log phase. From these cultures, the turbidity of cell suspensions were adjusted to an equivalent 0.5 McFarland standard as measured by absorbance (0.08–0.1 at 625 nm), corresponding to approximately 10^8^ CFU/ml. The adjusted cell suspensions were diluted 1:100 in double strengthen Mueller Hilton broth, and 50 μl was inoculated into each well (a final cell density ~ 5 × 10^5^ CFU/ml) in a microplate. The inoculum density was confirmed by plate count. Appropriate dilutions of the LysABP-01 in PBS (25 μl) were added into wells, and the same volume of PBS or antibiotic (0.25 × the MIC) was added. Sample-inoculated microplates were incubated at 37°C overnight. After incubation, 25 μl of 0.1% sterilized TTC (Sigma Chemical Co, Saint Louis, MO, USA) was added into each well and incubated for additional 3 h. The absorbance was read at 540 nm using a Synergy 2 multi-mode microplate reader (BioTek Instruments, Winooski, VT, USA). Each test was performed in triplicate.

### Checkerboard Synergy Testing

Interactions between LysABP-01 and the selected antibiotic were assessed by the checkerboard broth microdilution method as previously described (Pillai et al., 2005). Testing was performed in Mueller-Hinton broth, with a final inoculum of 5 × 10^4^ CFU per well in microplates. LysABP-01 and the selected antibiotic were diluted twofold horizontally and vertically, respectively. The concentrations of the selected antibiotic and LysABP-01 ranged from 0.0625 – 2 × MIC and 0.0156 – 2 × MIC, respectively. Microplates were incubated overnight at 37°C. After incubation, 25 μl of 0.1% sterilized TTC (Sigma Chemical Co, Saint Louis, MO, USA) was added into each well and incubated for 3 h. Test results were used to calculate the fractional inhibitory concentrations (FICs) and FIC index (FICI). FICs of LysABP-01 and the selected antibiotic were plotted on the x/y plot to generate an isobologram. A FICI was interpreted as follows: ≤0.5, synergy; >0.5 – ≤1.0, additive; >1.0 – ≤ 2.0, indifference; and >2.0, antagonism (Pillai et al., 2005).

## Results

### Characterization of the LysABP-01

**FIGURE 1:**
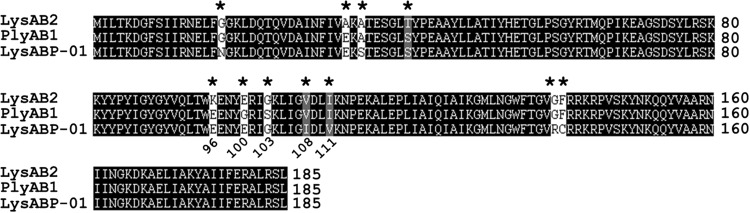
**Amino acid sequence alignment of LysABP-01.** The sequences used for alignment analysis were LysAB2 (from phage ϕAB2, accession no. ADX62345), PlyAB1 (from phage Abp1, accession no.YP_008058242) and LysABP-01 (from phage ØABP-01, accession no. AHG30899). Eleven mutation residues are indicated by asterisks (*), five of which are found in conserved domain at positions 96, 100, 103, 108, and 111.

The endolysin gene of the phage ØABP-01, named *lysABP-01*, has been previously detected and sequenced (accession no. KF548002). The LysABP-01 contains 185 amino acid residues, which corresponds to a 21.1 kDa protein. Conserved domain analysis using the Pfam database has revealed the presence of a lysozyme-like (*N*-acetyl-β-D-muramidase) catalytic domain between residues 75 and 128 of LysABP-01. A ClustalW2 alignment of LysABP-01 with two phage endolysins showed high similarity in the conserved domain region (**Figure 1**). Although these three endolysins share high sequence similarity, 5 out of 11 amino acid mutations were found in the conserved region among them (**Figure 1**).

### Over-Expression, Purification, and Lytic Activity of LysABP-01

**FIGURE 2:**
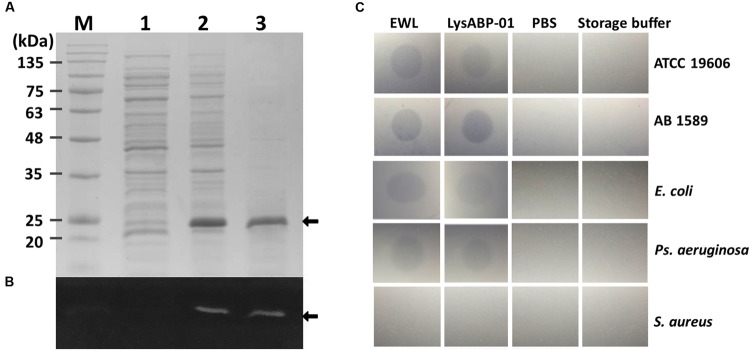
**Analysis of histidine-tagged LysABP-01. (A)** SDS-PAGE analysis for expression and purification of LysABP-01. **(B)** Fluorescent stain for detection of His_6_-tagged LysABP-01 on a SDS-PAGE gel. Lane M, BLUeye prestained protein ladder; Lanes 1, un-induced bacterial lysate; Lane 2, IPTG-induced bacterial lysate; Lane 3, the purified Lys-ABP-01 after dialysis. **(C)** Plate lysis assay of the purified LysABP-01. Egg white lysozyme (EWL), *A. baumannii* ATCC19606 (ATCC 19606), *A. baumannii* AB 1589 (AB 1589), *E. coli* (ATCC 25922), *Ps. aeruginosa* (ATCC 27853) and *S. aureus* (ATCC 6538).

After induction with IPTG, the expressed LysABP-01 was detected by SDS-PAGE analysis (**Figure 2A**). The presence of a histidine-tag in the expressed recombinant proteins was confirmed by staining with the Pierce 6xHis Protein Tag Stain Reagent Set (**Figure 2B**). We found that most of the expressed LysABP-01 was insoluble protein. The insoluble protein fraction was solubilized and purified using affinity chromatography. The purified LysABP-01 was refolded and concentrated. The lytic activity and spectrum of LysABP-01 were tested using the plate lysis assay and autoclaved cells (crude cell wall) from the log phase cells of gram-positive and gram-negative bacteria, which were used as substrates. The results showed that LysABP-01 had strong lytic activity toward two *A. baumannii* strains (ATCC 19606 and AB 1589) and weaker activity against reference strains of *E. coli* and *Ps. aeruginosa* (**Figure 2C**). In addition, no lysis was detected in a gram-positive strain, *S. aureus* (**Figure 2C**).

### Antibacterial Activity of LysABP-01 and Its Synergism with Antibiotics

**FIGURE 3:**
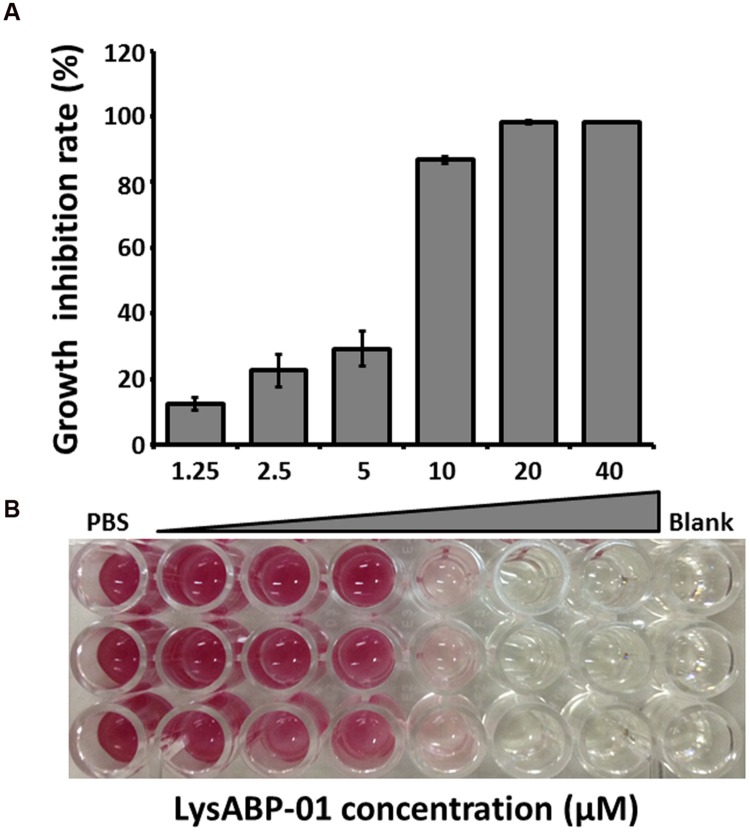
**Antibacterial activity of LysABP-01. (A)** Bacterial growth inhibition activity of varying concentrations of LysABP-01. The bar graph depicts the percent growth inhibition and represents the mean percentage ± SD of triplicate experiments. **(B)** The representative microplate image of TTC staining.

**FIGURE 4:**
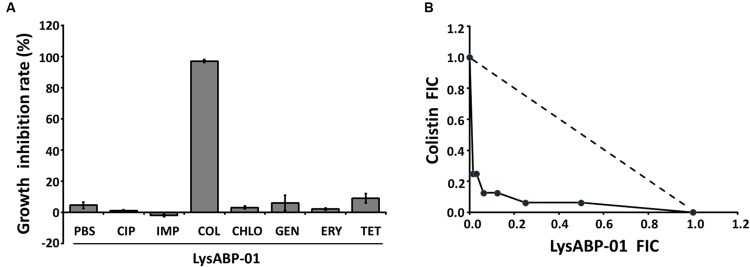
**The results of screening and confirmatory testing for synergistic interaction. (A)** The growth inhibition rate (the bar graph) of LysABP-01 combined with seven antibiotics. Data are expressed as the mean percentage ± SD of triplicate experiments. Phosphate buffer saline (PBS), ciprofloxacin (CIP), imipenem (IMP), colistin (COL), chloramphenicol (CHLO), gentamycin (GEN), erythromycin (ERY) and tetracycline (TET). **(B)** The isobologram of the checkerboard assay. FIC values derived from combinations of LysABP-01 and colistin were used to plot the isobologram. The dashed line illustrates the theoretical additive interaction between two agents.

The MIC of purified LysABP-01 was tested using bacterial growth inhibition assays for its ability to inhibit the growth of viable cells of the MDRAB strain. The results indicated that the LysABP-01 can prevent the growth of AB1589 with a MIC of 20 μM (**Figure 3**; **Table 2**). The interactions between LysABP-01 and seven antibiotics were screened by growth inhibition assay at 0.25 × the MIC of two agents (**Supplementary Table S1**). As shown in **Figure 4A**, only the combination of LysABP-01 plus colistin exhibited elevated antibacterial activity (nearly 100% of growth inhibition rate). In order to verify these results, we performed a checkerboard assay for measuring antibiotic synergy. The FICI value for the combination of LysABP-01 and colistin (0.0625/0.125 × the MIC) was determined as 0.188, which indicates synergism (**Table 2**). Test results were also represented by the isobologram generated by plotting the FICs of LysABP-01 and colistin. The isobologram is presented in **Figure 4B** and it shape being concave, suggests that the two antimicrobials had a synergistic effect against the representative MDRAB strain.

### The Susceptibility of XDRAB Strains to LysABP-01 Combined with Colistin

The selected strains of *A. baumannii* were obtained from various hospitals located in different areas of Thailand. All four isolates were positive for the species-specific *bla*_OXA-51_ gene (data not shown). The results from the disk diffusion method revealed that four *A. baumannii* isolates were resistant to all tested antibiotics except colistin and tigecycline. Thus, these strains were classified as XDRAB (**Table 2**). The *in vitro* activity of LysABP-01 against a broad range of *A. baumannii* strains was studied. The results of the antimicrobial susceptibility testing of different *A. baumannii* strains to LysABP-01 alone and in combination with colistin are shown in **Table 2** and **Supplementary Figure S1**. By a checkerboard test, the FICI and isobologram of the colistin-LysABP-01 combination showed synergistic against all tested XDRAB strains (**Table 2**; **Supplementary Figure S1**).

## Discussion

The emergence of drug-resistant bacteria is a therapeutic problem. Bacteriophages and their endolysins have been recognized as alternative therapeutic compounds for combating drug-resistant bacterial infections. In our previous work, three phages infecting MDRAB were isolated and characterized (Kitti et al., 2014). We found the ØABP-01 phage belonged to *Podoviridae* family. This phage had shown a good lytic activity on MDRAB and its genome contains the endolysin gene, named *lysABP-01*, which was further studied. *In silico* sequence analysis revealed that the LysABP-01 was conserved in other endolysins from Acinetobacter phage such as LysAB2, PlyAB1 and ABgp46 (Lai et al., 2011; Huang et al., 2014; Oliveira et al., 2016). The lysozyme-like catalytic domain of LysABP-01 is a *N*-acetyl-β-D-muramidase that cleaves β-1, 4-glycosidic bonds between *N*-acetylmuramic acid and *N*-acetylglucosamine of the peptidoglycan (Schmelcher et al., 2012). As illustrated in **Figure 1**, 11 mutation residues among three endolysins, especially five points in the conserved domain, may contribute to the enzyme’s catalytic function.

The recombinant LysABP-01 was located in the insoluble fraction as inclusion bodies due to its toxicity to host cells (García et al., 2010). We have successfully purified and refolded LysABP-01 from inclusion bodies. The result from plate lysis assay revealed that the recombinant LysABP-01 was able to digest crude cell wall from *A. baumannii* reference and MDRAB strains. This result was consistent with the findings from conserved domain analysis. However, LysABP-01 was active against other gram-negative bacteria but not able to hydrolyze crude cell walls of gram-positive bacteria, which corresponded with previous reports (Lim et al., 2014; Oliveira et al., 2014).

Enzyme activity of LysABP-01 was not only active against crude cell wall but also able to inhibit the growth of viable cells. The antibacterial activity of LysABP-01 was in agreement with the report of Lai and co-workers (Lai et al., 2011). In their study, they found that the presence of 500 μg/ml of the LysAB2 could reduce the cell viability of *A. baumannii* to less than 1% (Lai et al., 2011), while in our study we found that the presence of 20 μM (~500 μg/ml) of LysABP-01 inhibited the growth of *A. baumannii*. Gram negative bacteria have an OM that can protect peptidoglycan from the direct contact of lytic enzymes. Thus, the combination approach can be used to increase the efficacy of lytic enzymes. Previous studies have reported that the combination of colistin with other antimicrobials, such as vancomycin (Gordon et al., 2010), teicoplanin (Wareham et al., 2011), and daptomycin (Galani et al., 2014) produced synergistic effects against MDRAB strains. Because only the combination of LysABP-01 and colistin was found to increase antibacterial activity (**Figure 4B**), the checkerboard assay was not carried out in all combinations. The *in vitro* combination of colistin with LysABP-01 in this study revealed a synergistic interaction against MDRAB, AB 1589. We also found that this protein is effective against a broad range of XDRAB for both exclusive and in combination treatments. Moreover, the MICs of LysABP-01 were reduced up to 32-fold, while the MICs of colistin were reduced up to eightfold in the combination (**Table 2**). Colistin, also called polymyxin E, is a cyclic lipopeptide antibiotic consisting of a cationic peptide ring and a lipophilic fatty acid tail. The positively charged molecules of this drug interact with the negatively charged lipid A phosphates and displace the divalent cations (Mg^2+^ and Ca^2+^) in the lipopolysaccharide, which is essential for the OM stability. This interaction causes OM damage and leakage of cellular components leading to cell death (Falagas and Kasiakou, 2005). The cell membrane destabilizing activity of colistin could be exploited to promote the penetration of the endolysin through the OM toward its target in the cell wall. Although polymyxins are often the most effective drugs for MDRAB and XDRAB treatment, the use of these drugs in clinical practice is very restricted because of their effects on resistance development and nephrotoxicity or neurotoxicity (Falagas and Kasiakou, 2005; Cai et al., 2012). In this work, we have demonstrated that the colistin-LysABP-01 combination can produce synergy with a wide range of strains, reduce the dose of two agents, and may decrease the toxicity of colistin. Thus, the synergistic results illustrate the potential for future therapeutic use of this combination.

## Conclusion

This study shows that LysABP-01 contains antibacterial activity against *A. baumannii.* This enzyme has a synergistic interaction with colistin, an antibiotic with a directed action against the bacterial cell membrane. The implication of this study is that LysABP-01 will work as an alternative agent in combination with colistin against *A. baumannii*.

## Author Contributions

Study conception and design: DK, and SS. Acquisition of data: RT. Analysis and interpretation of data: RT, and TK. Drafting of manuscript: RT, TK, and SS. Critical revision: RT, TK, and SS.

## Conflict of Interest Statement

The authors declare that the research was conducted in the absence of any commercial or financial relationships that could be construed as a potential conflict of interest.

## Supplementary Material

FIGURE S1 ***In vitro* synergistic activity of LysABP-01 combined with colistin against XDRAB strains. (A)** The representative checkerboard plates. **(B)** The isobolograms representing the drug interaction of LysABP-01 plus colistin. FIC values derived from combinations of LysABP-01 and colistin were used to plot the isobologram. The dashed line illustrates the theoretical additive interaction between two agents.

TABLE S1 **MICs of conventional antibiotics against *A. baumannii* AB 1589**.

## References

[B1] Briers Y. Cornelissen A. Aertsen A. Hertveldt K. Michiels C. W. Volckaert G. (2008). Analysis of outer membrane permeability of *Pseudomonas aeruginosa* and bactericidal activity of endolysins KZ144 and EL188 under high hydrostatic pressure. *FEMS Microbiol. Lett.* 280 113–119. 10.1111/j.1574-6968.2007.01051.x 18248421

[B2] Briers Y. Walmagh M. Lavigne R. (2011). Use of bacteriophage endolysin EL188 and outer membrane permeabilizers against *Pseudomonas aeruginosa*. *J. Appl. Microbiol.* 110 778–785. 10.1111/j.1365-2672.2010.04931.x 21241420

[B3] Brown S. Young H. K. Amyes S. G. (2005). Characterisation of OXA-51, a novel class D carbapenemase found in genetically unrelated clinical strains of *Acinetobacter baumannii* from Argentina. *Clin. Microbiol. Infect.* 11 15–23. 10.1111/j.1469-0691.2004.01016.x 15649299

[B4] Cai Y. Chai D. Wang R. Liang B. Bai N. (2012). Colistin resistance of *Acinetobacter baumannii*: clinical reports, mechanisms and antimicrobial strategies. *J. Antimicrob. Chemother.* 67 1607–1615. 10.1093/jac/dks084 22441575

[B5] Clinical and Laboratory Standards Institute [CLSI] (2014). *Performance Standards for Antimicrobial Susceptibility Testing; Twenty-Fourth Informational Supplement. CLSI Document M* 100-S24 Wayne, PA: Clinical and Laboratory Standards Institute.

[B6] Djurkovic S. Loeﬄer J. M. Fischetti V. A. (2005). Synergistic killing of *Streptococcus pneumoniae* with the bacteriophage lytic enzyme Cpl-1 and penicillin or gentamicin depends on the level of penicillin resistance. *Antimicrob. Agents Chemother.* 49 1225–1228. 10.1128/AAC.49.3.1225-1228.2005 15728935 PMC549239

[B7] Donovan D. M. Foster-Frey J. Dong S. Rousseau G. M. Moineau S. Pritchard G. (2006). The cell lysis activity of the *Streptococcus agalactiae* bacteriophage B30 endolysin relies on the cysteine, histidine-dependent amidohydrolase/peptidase domain. *Appl. Environ. Microbiol.* 72 5108–5112. 10.1128/AEM.03065-05 16820517 PMC1489305

[B8] Falagas M. E. Kasiakou S. K. (2005). Colistin: the revival of polymyxins for the management of multidrug-resistant gram-negative bacterial infections. *Clin. Infect. Dis.* 40 1333–1341. 10.1086/429323 15825037

[B9] Fischetti V. A. (2010). Bacteriophage endolysins: a novel anti-infective to control gram-positive pathogens. *Int. J. Med. Microbiol.* 300 357–362. 10.1016/j.ijmm.2010.04.002 20452280 PMC3666336

[B10] Galani I. Orlandou K. Moraitou H. Petrikkos G. Souli M. (2014). Colistin/daptomycin: an unconventional antimicrobial combination synergistic in vitro against multidrug-resistant *Acinetobacter baumannii*. *Int. J. Antimicrob. Agents.* 43 370–374. 10.1016/j.ijantimicag.2013.12.010 24560919

[B11] García P. Martínez B. Rodríguez L. Rodríguez A. (2010). Synergy between the phage endolysin LysH5 and nisin to kill *Staphylococcus aureus* in pasteurized milk. *Int. J. Food Microbiol.* 141 151–155. 10.1016/j.ijfoodmicro.2010.04.029 20537744

[B12] Gordon N. C. Png K. Wareham D. W. (2010). Potent synergy and sustained bactericidal activity of a vancomycin-colistin combination versus multidrug-resistant strains of *Acinetobacter baumannii*. *Antimicrob. Agents Chemother.* 54 5316–5322. 10.1128/AAC.00922-10 20876375 PMC2981237

[B13] Henwood C. J. Gatward T. Warner M. James D. Stockdale M. W. Spence R. P. (2002). Antibiotic resistance among clinical isolates of *Acinetobacter* in the UK, and in vitro evaluation of tigecycline (GAR-936). *J. Antimicrob. Chemother.* 49 479–487. 10.1093/jac/49.3.479 11864948

[B14] Huang G. Shen X. Gong Y. Dong Z. Zhao X. Shen W. (2014). Antibacterial properties of *Acinetobacter baumannii* phage Abp1 endolysin (PlyAB1). *BMC Infect. Dis.* 14:681 10.1186/s12879-014-0681-2 PMC4274762 25495514

[B15] Kitti T. Thummeepak R. Leungtongkam U. Kunthalert D. Sitthisak S. (2015). Efficacy of *Acinetobacter baumannii* bacteriophage cocktail on *Acinetobacter baumannii* growth. *Afr. J. Microbiol. Res.* 9 2159–2165. 10.5897/AJMR2015.7696

[B16] Kitti T. Thummeepak R. Thanwisai A. Boonyodying K. Kunthalert D. Ritvirool P. (2014). Characterization and detection of endolysin gene from three *Acinetobacter baumannii* bacteriophages isolated from sewage water. *Indian J. Microbiol.* 54 383–388. 10.1007/s12088-014-0472 25320435 PMC4186927

[B17] Knezevic P. Petrovic O. (2008). Colorimetric microtiter plate method for assessment of phage effect on *Pseudomonas aeruginosa* biofilm. *J. Microbiol. Methods.* 74 114–118. 10.1016/j.mimet.2008.03.005 18433900

[B18] Lai M. J. Lin N. T. Hu A. Soo P. C. Chen L. K. Chen L. H. (2011). Antibacterial activity of *Acinetobacter baumannii* phage ΦAB2 endolysin (LysAB2) against both gram-positive and gram-negative bacteria. *Appl. Microbiol. Biotechnol.* 90 529–539. 10.1007/s00253-011-3104-y 21264466

[B19] Lim J. A. Shin H. Heu S. Ryu S. (2014). Exogenous lytic activity of SPN9CC endolysin against gram-negative bacteria. *J. Microbiol. Biotechnol.* 24 803–811. 10.4014/jmb.1403.03035 24690638

[B20] Magiorakos A. P. Srinivasan A. Carey R. B. Carmeli Y. Falagas M. E. Giske C. G. (2012). Multidrug-resistant, extensively drug-resistant and pandrug-resistant bacteria: an international expert proposal for interim standard definitions for acquired resistance. *Clin. Microbiol. Infect.* 18 268–281. 10.1111/j.1469-0691.2011.03570.x 21793988

[B21] Navon-Venezia S. Leavitt A. Carmeli Y. (2007). High tigecycline resistance in multidrug-resistant *Acinetobacter baumannii*. *J. Antimicrob. Chemother.* 59 772–774. 10.1093/jac/dkm018 17353223

[B22] Oliveira H. Thiagarajan V. Walmagh M. Sillankorva S. Lavigne R. Neves-Petersen M. T. (2014). A thermostable *Salmonella* phage endolysin, Lys68, with broad bactericidal properties against gram-negative pathogens in presence of weak acids. *PLoS ONE* 9:e108376 10.1371/journal.pone.0108376 PMC4188523 25290100

[B23] Oliveira H. Vilas Boas D. Mesnage S. Kluskens L. D. Lavigne R. Sillankorva S. (2016). Structural and enzymatic characterization of ABgp46, a novel phage endolysin with broad anti-gram-negative bacterial activity. *Front. Microbiol.* 7:208 10.3389/fmicb.2016.00208 PMC4768612 26955368

[B24] Perez F. Hujer A. M. Hujer K. M. Decker B. K. Rather P. N. Bonomo R. A. (2007). Global challenge of multidrug-resistant *Acinetobacter baumannii*. *Antimicrob. Agents Chemother.* 51 3471–3484. 10.1128/AAC.01464-06 17646423 PMC2043292

[B25] Pillai S. K. Moellering R. C. Eliopoulos G. M. (2005). “Antimicrobial combinations,” in *Antibiotics in Laboratory Medicine* ed. Lorian V. (Philadelphia, PA: Lippincott, Williams and Wilkins) 365–440.

[B26] Schmelcher M. Donovan D. M. Loessner M. J. (2012). Bacteriophage endolysins as novel antimicrobials. *Future Microbiol.* 7 1147–1171. 10.2217/fmb.12.97 23030422 PMC3563964

[B27] Triantaphpyllopoulos D. C. Quick A. J. Greenwalt T. J. (1955). Action of disodium ethylenediaminetetracetate on blood coagulation; evidence of the development of heparinoid activity during incubation or aeration of plasma. *Blood* 10 534–544. 14363334

[B28] Wareham D. W. Gordon N. C. Hornsey M. (2011). In vitro activity of teicoplanin combined with colistin versus multidrug-resistant strains of *Acinetobacter baumannii*. *J. Antimicrob. Chemother* 66 1047–1051. 10.1093/jac/dkr069 21393131

[B29] Young R. (1992). Bacteriophage lysis: mechanism and regulation. *Microbiol. Rev.* 56 430–481. 1406491 10.1128/mr.56.3.430-481.1992 PMC372879

