# Supplementary figures and images for: Enhanced Antibacterial Activity of Acinetobacter baumannii Bacteriophage ØABP-01 Endolysin (LysABP-01) in Combination with Colistin
Source: Front Microbiol. 2016 Sep 7;7:1402. doi: 10.3389/fmicb.2016.01402 (PMC5013039; doi:10.3389/fmicb.2016.01402)

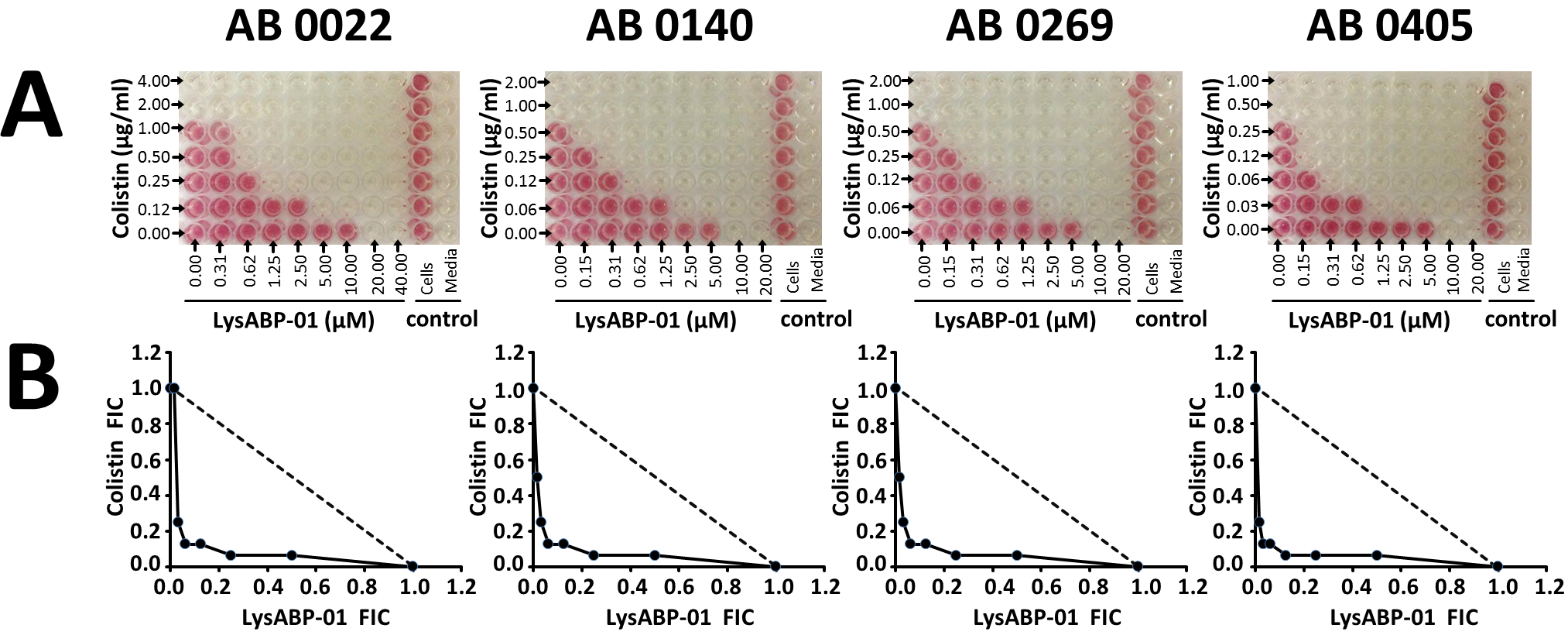

Supplement: FIGURE S1 — In vitro synergistic activity of LysABP-01 combined with colistin against XDRAB strains. (A) The representative checkerboard plates. (B) The isobolograms representing the drug interaction of LysABP-01 plus colistin. FIC values derived from combinations of LysABP-01 and colistin were used to plot the isobologram. The dashed line illustrates the theoretical additive interaction between two agents. [file Image_1.TIF]
