# Supplementary material for: Enhanced Antibacterial Activity of Acinetobacter baumannii Bacteriophage ØABP-01 Endolysin (LysABP-01) in Combination with Colistin
Source: Front Microbiol. 2016 Sep 7;7:1402. doi: 10.3389/fmicb.2016.01402 (PMC5013039; doi:10.3389/fmicb.2016.01402)
Supplement: TABLE S1 — MICs of conventional antibiotics against A. baumannii AB 1589. [file Table_1.DOCX]

**Supplemental data**

**Table S1. MICs of conventional antibiotics against *A. baumannii* AB 1589**

| **Antibacterial agents** | **MICs (µg/ml)** | |
| --- | --- | --- |
|  | **1 x MICs** | **0.25 x MICs** |
| Ciprofloxacin (CIP) | 16 | 4 |
| Imipenem (IMP) | > 256 | 64 |
| Colistin (COL) | 1 | 0.25 |
| Chloramphenicol (CHLO) | 32 | 8 |
| Gentamycin (GEN) | > 256 | 64 |
| Erythromycin (ERY) | > 256 | 64 |
| Tetracycline (TET) | 128 | 32 |
